# Supplementary material for: The Systems Biology Research Tool: evolvable open-source software
Source: BMC Syst Biol. 2008 Jun 29;2:55. doi: 10.1186/1752-0509-2-55 (PMC2446383; doi:10.1186/1752-0509-2-55)
Supplement: Additional file 1 — SBRT Archive. An archive of the current version of the Systems Biology Research Tool. [file 1752-0509-2-55-S1.zip › sbrt-1.4.0/doc/developers_guide/api/sbrt/kernel/math/algebra/LinearSystemSolver.html]

LinearSystemSolver


|  |  |  |
| --- | --- | --- |
| **Overview** \| **Package** \| **Class** \| **Use** \| **Tree** \| **Deprecated** \| **Index** \| **Help** | |  |
| **PREV CLASS**   **NEXT CLASS** | **FRAMES**    **NO FRAMES**     **All Classes** |  |
| SUMMARY: NESTED \| FIELD \| CONSTR \| METHOD | DETAIL: FIELD \| CONSTR \| METHOD |  |


---


## sbrt.kernel.math.algebra Interface LinearSystemSolver<V>

**Type Parameters:**: `V` - the variable type.

**All Known Implementing Classes:**: MathematicaSolver

---

``` public interface LinearSystemSolver<V> ```

This interface is used to represent classes that solve systems of linear
equations.

**Author:**
:   This interface was written and documented by Jeremiah Wright
    while in the Wagner lab.

---

| **Method Summary** | |
| --- | --- |
| `LinearSystemSolution<V>` | `solve(java.util.Map<LinearComb<V>,java.lang.Double> equations)`             Computes and returns a solution to the provided system of linear equations. |

**Method Detail**

### solve

```
LinearSystemSolution<V> solve(java.util.Map<LinearComb<V>,java.lang.Double> equations)
```

:   Computes and returns a solution to the provided system of linear equations.

    :   **Parameters:**: `equations` - linear combinations mapped to a double. Each key-value pair represents the left and right hand sides, respectively, of an equation. **Returns:**: a solution to the provided system of linear equations.


---


|  |  |  |
| --- | --- | --- |
| **Overview** \| **Package** \| **Class** \| **Use** \| **Tree** \| **Deprecated** \| **Index** \| **Help** | |  |
| **PREV CLASS**   **NEXT CLASS** | **FRAMES**    **NO FRAMES**     **All Classes** |  |
| SUMMARY: NESTED \| FIELD \| CONSTR \| METHOD | DETAIL: FIELD \| CONSTR \| METHOD |  |


---
